# The influence of specific cognitive training in virtual reality on the inhibition of elite young ice hockey players

**DOI:** 10.3389/fspor.2025.1682165

**Published:** 2025-10-30

**Authors:** Florian Heilmann, Torsten Schubert

**Affiliations:** ^1^ Movement Science Lab, Institute for Sport Science, Martin Luther University Halle-Wittenberg, Halle (Saale), Germany; ^2^ Experimental Psychology, Institute for Psychology, Martin Luther University Halle-Wittenberg, Halle (Saale), Germany

**Keywords:** executive functions, cognitive training, ice hockey, virtual reality, youth athletes

## Abstract

**Introduction:**

Executive functions (EFs) such as inhibition skills are crucial in sports, particularly in game sports, as they facilitate rapid decision-making, impulse control, and effective reactions to unforeseen situations. This study investigates the influence of specific cognitive training (CT) in virtual reality (VR) on inhibition in young ice hockey players compared to an individual technical training session. The potential implications of this research are significant, as it could lead to the development of new training methods to improve sports performance.

**Methods:**

The study involved 25 young ice hockey players (5 female, Mdn: 14 years, span: 11–17 years). Before and after the training period, the test subjects completed sport-specific and general tests to measure inhibition ability (Go/No go task, Flanker task, sport-specific modified using a special measuring station). The intervention group (*N* = 12) engaged in sport-specific CT in virtual reality (2 times/week; 9 weeks), and the control group (*N* = 13) completed individual technique training.

**Results:**

For the Cued GoNoGo task, no significant main effects could be determined for the specific and non-specific tests (reaction time, accuracy). For the flanker task, significant main effects were found for the sport-specific test for the congruent (pre-post: *p* < .001; int.: *p* < .001; group: *p* = .112) and incongruent (pre-post: *p* < .001; int.: *p* < .001; group: *p* = .105) but not for the flanker effect (pre-post: *p* = .364; int.: *p* < .526; group: *p* = .597).

**Discussion:**

The results show significant improvements in the flanker task for the intervention group in the sport-specific test for congruent and incongruent conditions. This suggests that CT in VR can potentially improve sport-specific inhibition skills in young ice hockey players, particularly in relation to dealing with distracting stimuli or distractors (flanker task). There were no prominent effects for domain-generic cognition tasks. Further research is needed to understand the long-term effects and the transferability of these training effects on ice hockey performance.

## 1 Introduction

In the 1980s, researchers began referring to frontal lobe functioning and mental control over lower-level cognitive functions as “executive function,” which led to the coining of the term executive function (EF) (1) in the neuropsychological literature (2). It is believed that EFs are essential to human cognition and behavior and that they are essential for determining the efficiency of goal-directed behavior, especially in dynamic contexts (3). While different EFs can be distinguished, inhibition as one of these functions has been shown to play a pivotal role in athletic performance, especially in dynamic and fast-paced sports like ice hockey (4, 5). Inhibition, the ability to control impulsive responses and focus on task-relevant stimuli, is essential for athletes to make quick decisions, adapt to changing circumstances, and maintain control under pressure (6). Friedmann and Miyake (3) describe the unity and diversity of the EFs. In the recent study there is a focus on inhibition. Nevertheless, the different functions could not be easily differentiated. In open-skill sports (7), game sports, where split-second decisions can determine success or failure, enhancing these cognitive abilities could provide a critical edge. However, up to now, the issue of whether focused cognitive training (CT) can improve the general ability of participants to perform well in these tasks remains open, such as the question of whether or not EFs can be improved by training (8).

With applications being thoroughly examined in various cohorts, computerized CT is a rapidly developing research field, which has strong implications also for the applied context (9). Through recurrent computer exercises, the main goal of CT is to improve certain cognitive functions by practice or training (8). There is an ongoing debate about the effectiveness of CT in different contexts (https://www.cognitivetrainingdata.org/the-controversy-does-brain-training-work/response-letter/ (10). Furthermore, studies in the field of CT were criticized for high methodological heterogeneity, a rather low ability to define improvements in a functional capacity, and minor sample sizes, etc., (11). Thus, Green et al. (12, 13) developed methodological standards for training studies, focusing on the potential improvement of cognitive functions by training interventions. Indeed, there are empirical reports and observations that imply that suitable training interventions can result in broad learning effects and may generalize to other non-trained tasks (14–16). On the other hand, it is important to distinguish this type of training from strategy-based training approaches, which typically concentrate on teaching task-specific techniques and skills that enhance performance in the assigned task without generalizing to other tasks [e.g., (17, 18)].

Nevertheless, there are current meta-analyses reporting a positive effect of CT on EFs for preschoolers (19, 20), adolescents, and adults (21) as well as older adults (22–25). Based on these findings, it is tempting to investigate whether EFs can be trained as experts respectively in athletes of different sports disciplines and which type of intervention gains appropriate effects. This leads to the research question of the current study, namely, to what extent EFs of youth competitive ice hockey players can be improved by CT intervention applying a special Virtual Reality (VR) setting as compared to a standard individual technical training session.

CT interventions are usually directed to improve a person's cognitive abilities and brain activity such that task performance can benefit (26). According to Mayer et al. (27) CT interventions encompass the active design of both thought and imagination processes such that performance-enhancing cognitions can be retrieved according to the situation. In particular, this includes CT interventions designed to improve performance in game scenarios within sports (e.g., ice hockey). The literature distinguishes domain-specific or sport-specific CT from domain-generic or domain-unspecific CT. This suggests applying cognitive abilities to situations that are near or far from the trained situation. A transfer of skills is the generalization of abilities or functions taught or trained in several contexts. Near transfer is the transmission of abilities between similar disciplines (or situations) with strong structural similarities of the relevant cognitive processes. Conversely, far transfer takes place in a weak or unconnected way between domains and is associated with the structural similarity between the tasks in the trained and in the transfer situation (28). The psychological literature is well aware that while far transfer is observed less often than near transfer, it is much more interesting to study (14, 16). However, research on the cognitive transfer of EFs in athletes exists, but the results are rather inconsistent (29), especially if the transfer of training to other laboratory tasks (30–32) or the transfer to real-world tasks is examined (33, 34).

Harris et al. (29) reported limited far transfer effects from CT to athletic tasks, likely due to differences between athletic and non-athletic environments. More substantial effects are expected when tasks closely align with sports contexts. For example, NeuroTracker training improves near transfer (multiple object tracking) but not broader perceptual-cognitive skills (35). While Heisler et al. (36) linked executive functions (EFs), particularly working memory, to sport-specific decision-making, evidence on whether sports foster EF development remains mixed. Beavan et al. (37, 38) found no clear link between football experience and EFs, and meta-analyses have reported contradictory results (7, 39). Similarly, Moen et al. (40) confirmed NeuroTracker's limited EF benefits, and Heilmann et al. (41) found no EF improvements from smartphone training (Fruit Ninja©) in soccer players. Ceiling effects (42) and limited adaptability in younger and middle-aged groups (21) may explain these outcomes. Overall, findings on EF interventions in athletes remain inconsistent.

Further studies examined the effect of more ecologically valid training (e.g., virtual reality training) on cognitive functions and applied training interventions based on virtual reality (VR) technology. By definition, VR is the “interactive visualization of virtual images enhanced by special processing and nonvisual display modes: to convince participants that they are immersed in a synthetic space” [(43), p.124]. Different technologies are available, which apply VR: head-mounted display (HMD), Cave Automatic Virtual Environment (CAVE), or exergaming (44).

Huang (45) compared an immersive CT (game Fruit Ninja; HMD) with a non-immersive CT in older adults, which could reveal significant differences in EFs (Stroop and Trail-making task) improvement concerning the presence of the CT. The findings of Grosprêtre et al. (46) show a significant improvement of EFs (inhibition, go/no-go, Stroop task) in young adults by VR training, but not by video training using shadow boxing fitness videos. Sañudo et al. (47) examined the effects of aerobic exercise with superimposed VR on young males’ EFs (cognitive flexibility and inhibition) and selective attention. They reported significant time reductions for all WCST and Stroop outcomes in the experimental group. Lachowicz et al. (48) examined the effects of VR training on concentration performance and EF (Color Trail making test) in E-athletes. Findings show significant effects for the experimental group (49). In the study of Fortes et al. (50) VR training significantly improved passing decision-making performance and visual search behavior of participants. In this study, inhibitory control was improved in both groups, the VR training group and a group applying a standard video-screen training, but without a group interaction effect (*p* > 0.05).

In sum, sport-specific CT is a promising avenue for improving cognitive skills; however, it should be combined with sport-specific assessments, as this considers the specific athletic environment [see (29)]. The initial actions in this regard have already been completed (51–53) and the current study is aimed at extending this research.

Montuori et al. (54) developed a sport-specific task-switching protocol in ice hockey, showing positional differences and suggesting value for player selection. Heilmann et al. (55) used SoccerBot360 to measure EFs in a 360° simulation, finding age-related development similar to standard EF tasks. Musculus et al. (53) and Knöbel & Lautenbach (52) validated soccer-specific inhibition and flexibility tasks with motor responses, demonstrating good reliability. The SoccerBot360 number-letter task showed valid switch effects, while the flanker task required modification due to weak convergent validity. Both were recommended as diagnostic tools, with similar findings for working memory tasks. There is a research gap regarding how sport-specific CT enhances EF and how sport-specific assessments are utilized to measure this improvement. In the present article, we report on the findings of an extensive investigation of the effects of domain-specific CT compared to a common individual technical training session on young ice hockey players’ EFs. For this purpose, we investigated a group of young ice hockey players by exposing them to a series of CT sessions specifically designed for on-ice decision-making and cognitively demanding virtual reality scenarios (nine weeks, twice a week). The CT aimed to improve cognitive flexibility and inhibitory control. A battery of EF tasks (domain-generic [PC] and domain-specific [specific stimuli and response) were used for pre-and post-intervention evaluations. The results are important because they can aid professionals in selecting whether to use VR or CT training in their work and, in addition, they may help practitioners and academics to assess the prospects and determine whether creating CT treatments or domain-specific measurements (EF) tailored to particular sports is advantageous (8). This is particularly relevant for ice hockey players, as the sport demands rapid decision-making under pressure, constant task-switching between offensive and defensive actions, and strong inhibitory control to avoid penalties or errors. We hypothesized that youth ice hockey players receiving domain-specific CT in VR would improve inhibition compared to the control group. Furthermore, we hypothesized that the improvements are more significant in the ecologically valid task.

## 2 Methods

### 2.1 Participants

**Figure 1:**
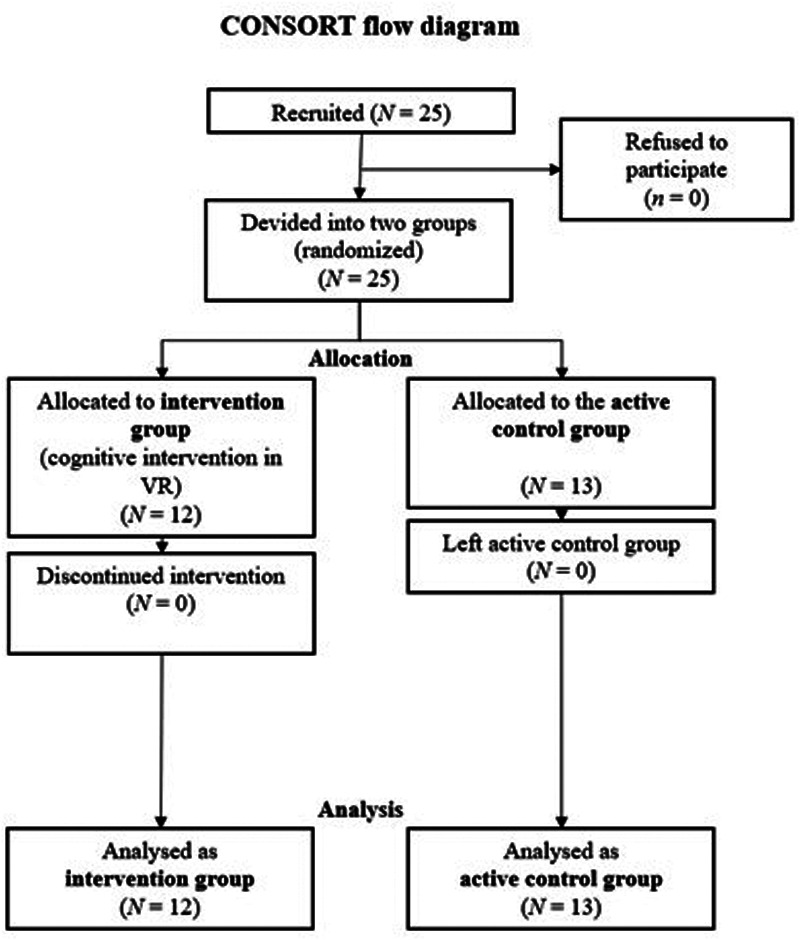
CONSORT flow diagram of randomization, allocation, and analysis of participants.

Twenty-five ice hockey players of a youth academy aged 11–17 (5 females, Mdn = 14) participated voluntarily in the study. The participants for organizational reasons, players from one training group were assigned to the intervention group (*n* = 12). The other group was assigned to the control group (*n* = 13) and had an individual technical skills training session with the same duration as the intervention group (Figure 1). There were no significant differences in performance or other relevant variables (such as age) before the intervention. The study protocol followed the Declaration of Helsinki and the APA's ethical guidelines. No formal visual acuity assessment was conducted prior to participation in the VR training tasks. However, all participants were members of a youth hockey academy, where routine medical screenings, including vision checks, are standard practice. It was therefore assumed that participants had normal or corrected-to-normal vision sufficient for the visual demands of the tasks. We did not conduct an *a priori* power analysis, as no previous studies with sufficiently comparable outcomes were available to provide a reliable basis for estimating the required sample size. The study was approved by the university's ethical committee (approval number of the ethical committee of Martin-Luther-University Halle-Wittenberg: 2024-151).

### 2.2 Measurements

**Figure 2:**
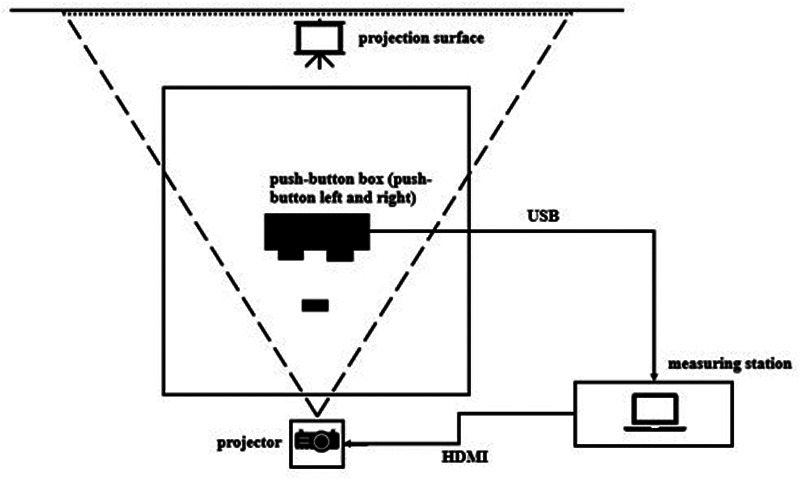
Measurement setting.

The EFs were measured using Inquisit Lab 6 (Millisecond Software LLC, Seattle, WA, USA) on a 17-inch screen, a QWERTZ keyboard (PC [personal computer), and a self-developed measuring device (see Figure 2; IH [ice hockey). The modified tasks were displayed using a projector (Epson EB-L630U, Seiko Epson Corporation, Nagano, Japan). The responses were given with two push buttons covered with foam so the participants could hit them with their hockey sticks.

**Figure 3:**
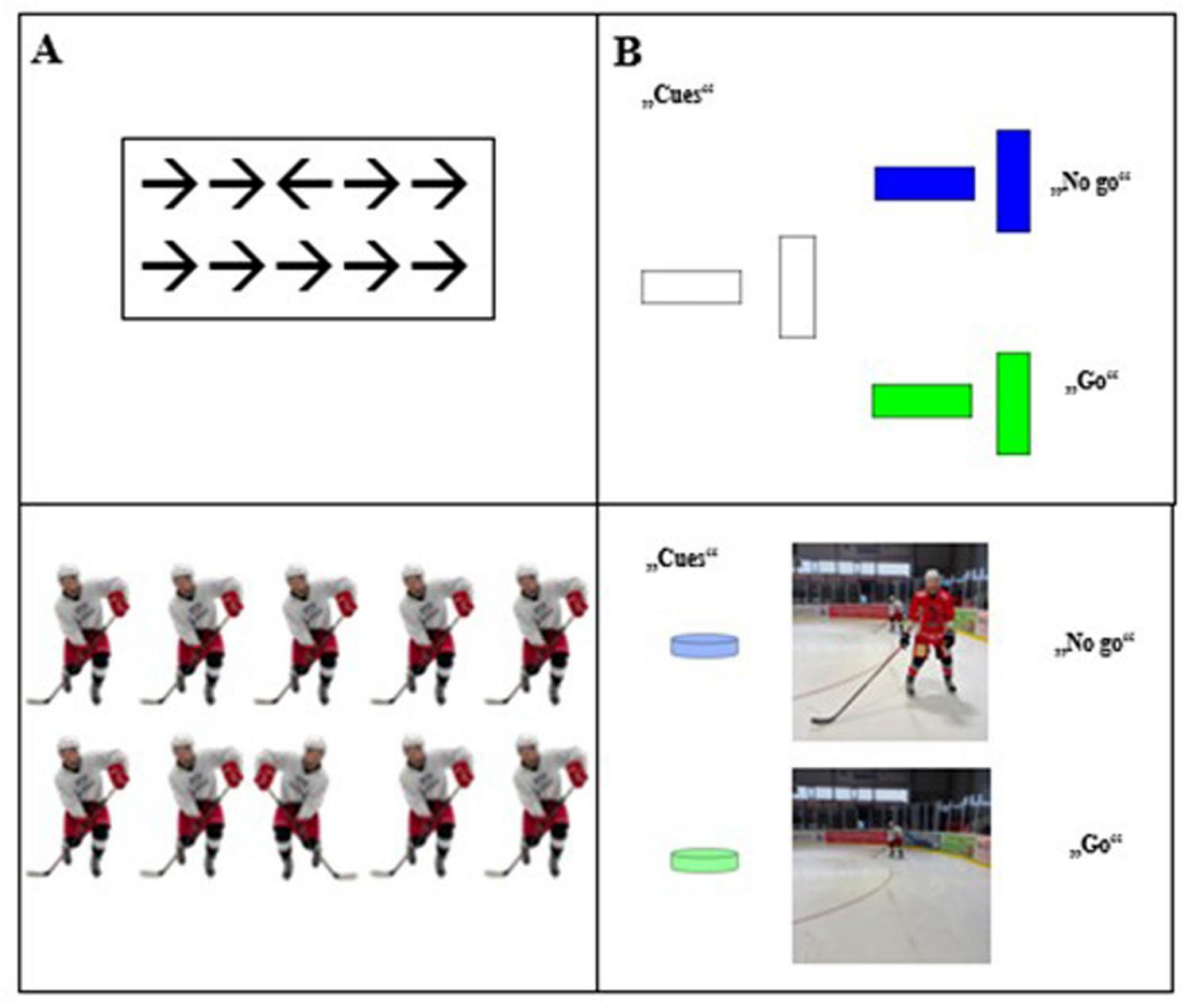
Stimulus material, **(A)** Flanker task; **(B)** Cued Go/No Go task (upper part: standard version; lower part: modified version).

For the Flanker task (see Figure 3), participants were shown a stimulus with five black arrows on a white background, with a central “target arrow” to which they responded. In congruent trials, all arrows are pointed in the same direction, while in incongruent trials, the central arrow is pointed in the opposite direction to the surrounding flanker arrows. Participants pressed the right button if the target arrow pointed right and the left button if it pointed left (4 practice trials, 70 test trials). In the Go/No-Go task, participants were instructed to press the space key when shown a green rectangle (Go) but when shown a blue rectangle (No-Go). These rectangles can appear either vertically (positive cue, the likelihood for a following green rectangle is 80%) or horizontally (negative cue, the likelihood for the blue rectangle is 80%). Fifty trials were conducted. In the modified tasks, the rectangles of the Go/No go task were replaced by a picture illustrating an ice hockey situation with the opportunity to pass a puck or with an opponent blocking the pass way, and the arrows of the Flanker task were replaced by teammates allowing passing the puck to their left or right side (see Figure 1; with their stick on the left or right side). Additionally, the Flanker effect was used to quantify the impact of stimulus incongruency on the difference in response times between congruent and incongruent stimuli. The smaller the size of the Flanker effect, the better the inhibitory control in the participant. This approach has provided good reliability coefficients in earlier studies (56); r = 0.856 for congruent trials [response time] and r = 0.879 for incongruent trials [response time).

### 2.3 Intervention

VR training intervention: A sport-specific, respectively, ice hockey-specific CT intervention was developed by searching for various exercises of the software SenseArena (Sense Arena S.R.O., Praha, Czech Republic) adapted for training cognitive functions (EFs) and especially for inhibition. The training was conducted using a head-mounted display (e.g., Meta Quest), along with a controller mounted on the players’ hockey sticks to track stick movements. Players were checked for dizziness and cyber sickness before first procedures. The intervention took place in a quiet room at the club's training facility, with players wearing regular sports attire and using the Meta Quest 2 headset to interact with the Sense Arena software. The players were familiarized with the VR situation by being allowed to spend 20 min in a simple virtual environment. They had no experience with virtual environments before the familiarization or before the intervention.

The exercises were implemented in a 9-week training plan, consisting of two training units per week, each with a duration of 40–60 min (2 × 20–30 min per training unit).

During the virtual reality (VR) training protocol, participants completed three structured drills selected from the categories Offensive Zone, Advanced Drills, and Cognitive. The drills—Multiple-Object-Tracking (MOT), Find the Lane, and Divided Attention—were chosen for their focus on cognitive control, attentional flexibility, and decision-making under time pressure.

The Multiple-Object-Tracking (MOT) task required participants to simultaneously monitor several designated targets among multiple moving players while maintaining puck control. In addition to dynamic tracking, players were briefly exposed to color cues that needed to be remembered and recalled later, introducing a working memory component. This task was designed to enhance selective attention, information updating, and the ability to sustain performance in environments with high visual and cognitive load, closely simulating the perceptual demands of in-game play. By requiring participants to ignore irrelevant distractors while selectively focusing on designated targets, this drill specifically trains inhibitory control in dynamic visual contexts. In the Find the Lane drill, participants were placed in an offensive scenario requiring the execution of a pass through a dynamically moving target to a teammate. Task difficulty was manipulated through variations in target movement speed, angular displacement, and passing distance. The drill was intended to develop anticipatory decision-making, spatial awareness, and motor precision. By forcing participants to adapt to temporal and spatial constraints, the task replicated the cognitive-perceptual requirements of offensive playmaking in ice hockey. Inhibitory control is engaged here as players must suppress prepotent but suboptimal passing options, delaying action until the correct lane emerges, thereby enhancing the ability to inhibit premature responses. The Divided Attention drill presented a dual-task paradigm, in which participants were required to identify matching symbols within a grid of distractors while concurrently performing puck-handling tasks. This condition imposed competing cognitive demands, requiring the filtering of irrelevant stimuli and the flexible allocation of attentional resources across tasks. The exercise targeted executive control functions, particularly inhibitory processing, cognitive flexibility, and the capacity to sustain accuracy in multitasking contexts. Inhibitory control is directly trained through the suppression of irrelevant or misleading stimuli in the symbol-matching task while maintaining accurate performance on the concurrent motor activity.

Collectively, these three drills operationalized core cognitive constructs relevant to high-performance hockey: tracking and updating (MOT), anticipatory decision-making under spatiotemporal constraints (Find the Lane), and divided attention with inhibitory control (Divided Attention). The structured use of VR provided an ecologically valid yet controllable environment, enabling the systematic training and assessment of players’ cognitive-perceptual skills, which are critical for on-ice performance. Control intervention: In contrast to the intervention group, the control group did not undergo any training tailored to cognitive aspects. Instead, it carried out standard individual technique training. In this case, individual means that the specific needs of the players were addressed in the corresponding training sessions. The technique training included stick handling, passing, and inline skate training and had the same number of training sessions as the VR intervention (9 weeks, 2 times per week, 20–30 min).

### 2.4 Procedures

All participants and their legal representatives gave their informed assent and consent. The informed consent form was given by participants after they had been briefed on the procedure. The participants underwent pre-and post-tests (EF-tests) at the movement science lab. The EF tasks (domain-generic or sport-specific) were conducted in a randomized order. To minimize the effects of physical exertion, the players were assessed between 10:00 a.m. and 4:00 p.m., one hour before training. Furthermore, the tests were conducted at the same time (+/- one hour) for the pre- and post tests. The experimenter gave the subjects an explanation of the intervention following the pretest. The participants had to fill out a questionnaire as well.

### 2.5 Data preparation

A preliminary filter for the Flanker task for inhibition eliminated all trials with erroneous responses (PC: pre: 1.9%; post: 2.7%; IH: pre: 1.1%; post: 2.1%). To accommodate extreme values, a second filter was applied (PC and IH for pre and post: ∼0.1%) to exclude all trials with response times lower than 200 ms or more than 1.750 ms [e.g., (53)] A final filter (PC: pre: 1.1%; post: 0.9%; IH: pre: 1.4%; post: 0.9%) and responses that were + - 3 SD outside the individual response time mean. For the Go/No go task the following portion of trials were excluded by the three filters: first: PC: pre: 1.0%; post: 1.3%; IH: pre: 1.1%; post: 1.7%; second: PC: pre: 2.0%; post: 1.8%; IH: pre: 1.0%; post: 2.3%; third: PC: pre: 2.0%; post: 1.1%; IH: pre: 1.4%; post: 1.8%. We had to reduce the total dataset because of the filtered datasets.

### 2.6 Statistical analysis

The analysis of variance (MANOVA) was used even though our data was not normally distributed because the procedure is resistant to violated normality test assumptions [e.g., (57)]. There were no outliers with more than 1.5 interquartile ranges to exclude.

To assess the effects of the intervention on inhibition, we conducted a 2 (time: pre vs. post) × 2 (test condition: PC [personal computer] vs. IH [ice hockey) × 2 (group: IG [intervention group] vs. CG [control group) multivariate analysis of variance (MANOVA) for response time parameters of the Go/No go task and flanker task (i.e., response time for incongruent trial) in determining whether inhibition altered over time as a result of the intervention and if the test condition has got a significant impact on the effect. Furthermore, we conducted separate 2 (time: pre vs. post) × 2 (test condition: PC [personal computer] vs. IH [ice hockey) × 2 (group: IG vs. CG) MANOVAs for accuracy measures for the Go/No go task and Flanker task because they were not linked with response time characteristics.

After that, we performed a *post-hoc* analysis using the Bonferroni correction process (univariate ANOVA and t-tests). SPSS 30 (SPSS, Chicago, Illinois, United States) was used for the statistical analysis. The threshold for significance was fixed at *p* < .05.

## 3 Results

### 3.1 Response time

**Figure 4:**
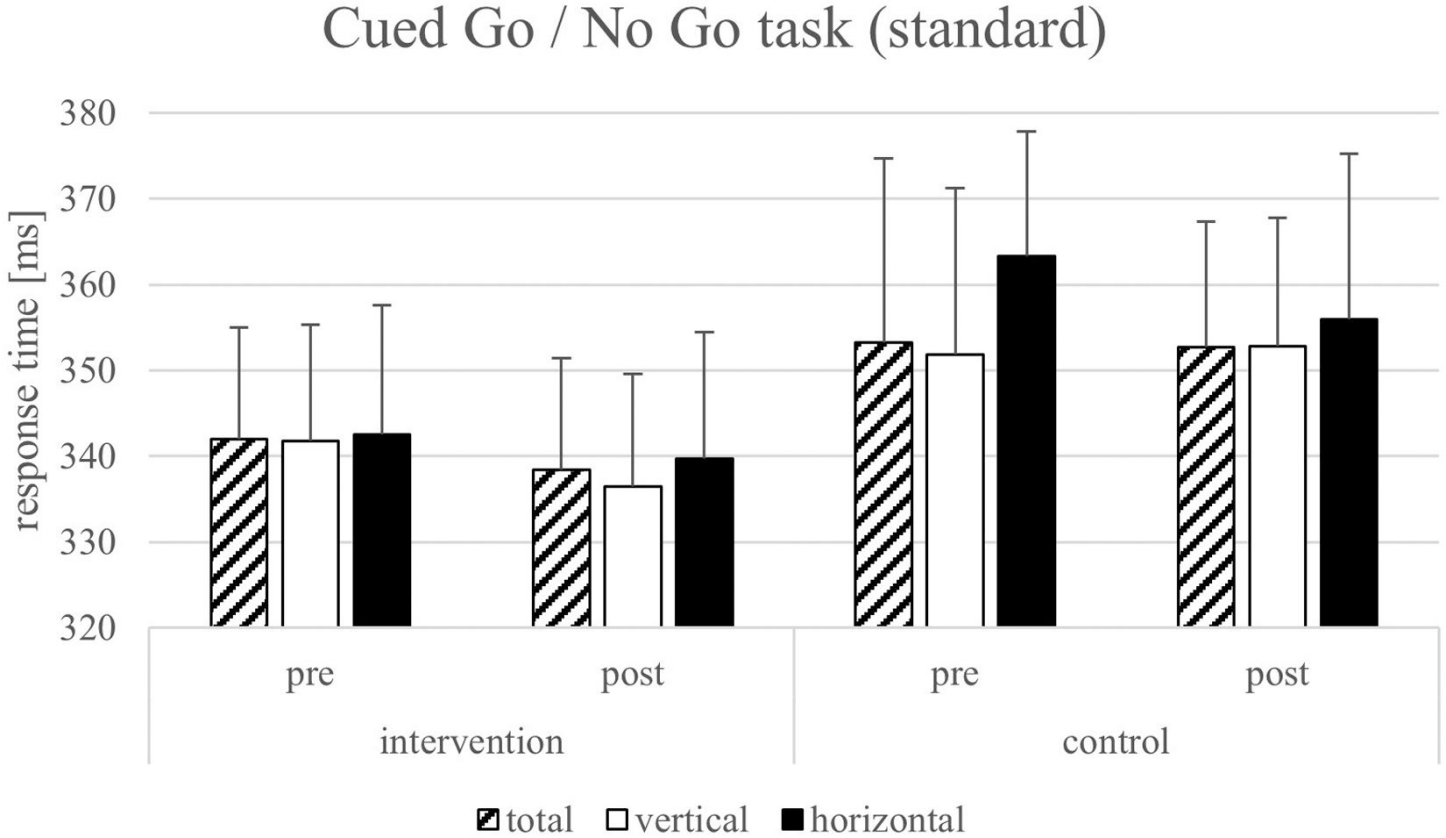
Response times [ms] and SEM for the Cued Go/No Go task (standard version).

**Figure 5:**
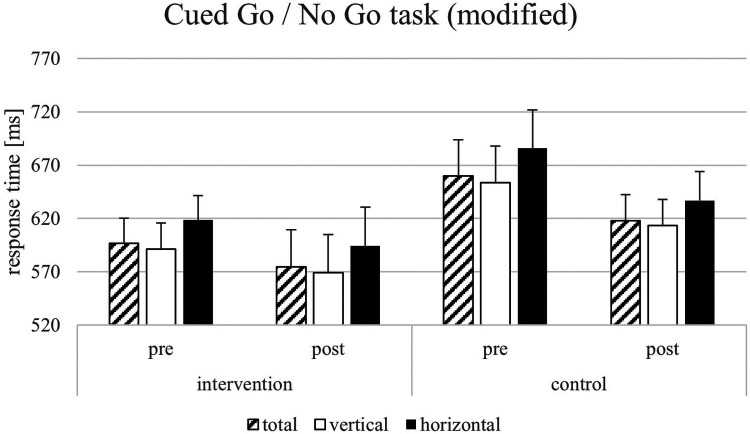
Response times [ms] and SEM for the Cued Go/No Go task (modified version).

**Figure 6:**
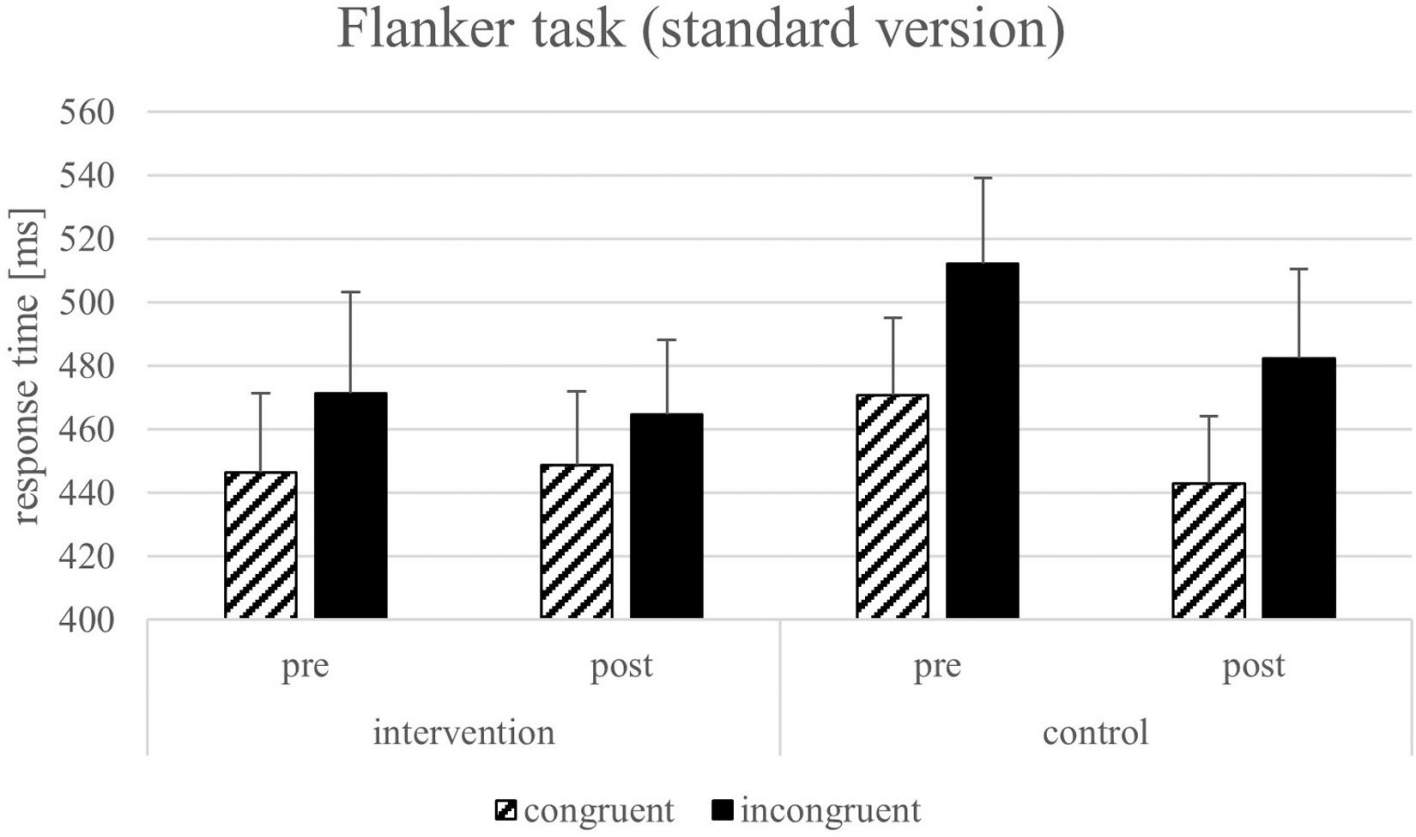
Response times [ms] and SEM for the Flanker task (standard version).

**Figure 7:**
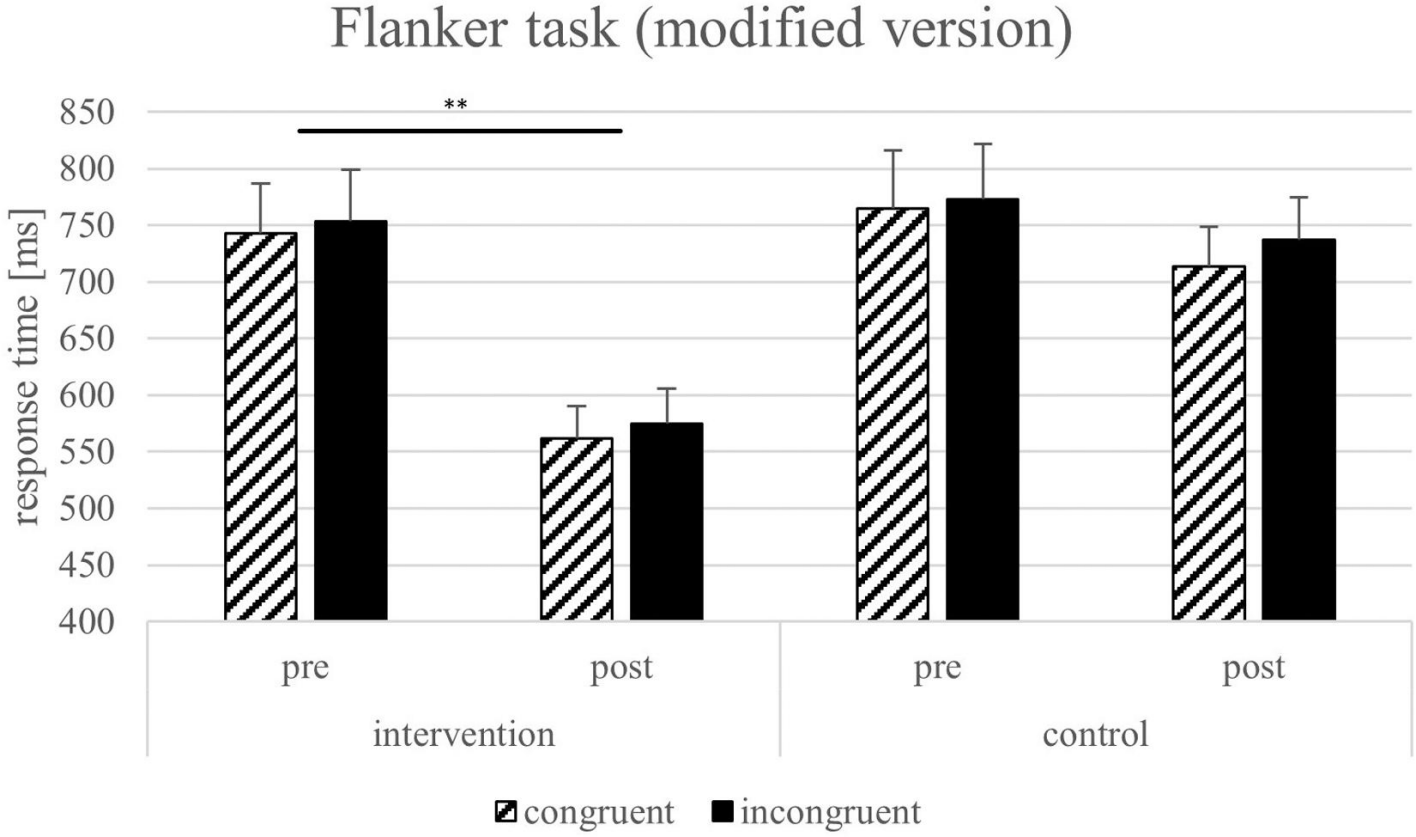
Response times [ms] and SEM for the Flanker task (modified version; *p = ≤ 0.05, **p = ≤ 0.01).

For the response time parameters of the Flanker task, the results showed significant main effects on the response times in congruent trials by the factors time [*F*(1, 22) = 20.07, *p* = < .001, *η*_p_2 = 0.722, 1-*β* = 0.964] but not of the factor group [*F*(1, 22) = 1.43, *p* = .247, *η*_p_2 = 0.067, 1-*β* = 0.069]. Furthermore, results showed significant effects on response times in incongruent trials by the factors time [*F*(1, 22) = 11.75, *p* = .003, *η*_p_2 = 0.370, 1-*β* = 0.299], test condition [*F*(1, 22) = 218.314, *p* = < .001, *η*_p_2 = 0.640, 1-*β* = 0.840] but not of the factor group [*F*(1, 22) = 1.918, *p* = .181, *η*_p_2 = 0.088, 1-*β* = 0.077]. For the Flanker effect, only the factor test condition was significant [*F*(1, 22) = 5.524, *p* = .029, *η*_p_2 = 0.216, 1-*β* = 0.524]. All other factors did not show significant effects (see Figures 4–6). The Flanker task results showed that response times in both congruent and incongruent trials were significantly influenced by time, test condition, and their interactions with group, but not by group alone. For the Flanker effect, only test conditions showed a significant effect. For the response time parameters of the Go/No go task, the results showed a significant effect of the test condition on the response times [*F*(1, 22) = 286.434, *p* = < .001, *η*_p_2 = 0.916, 1-*β* = 1], the interaction test condition*group [*F*(1, 22) = 1.112, *p* = .050, *η*_p_2 = 0.179, 1-*β* = 0.121], and test condition*time [*F*(1, 22) = 3.624, *p* < .001, *η*_p_2 = 0.640, 1-*β* = 0.840] but not for time*group [*F*(1, 22) = 0.230, *p* = .637, *η*_p_2 = 0.208, 1-*β* = 0.138]. For the response times in vertical trials, the results showed significant effects for test condition [*F*(1, 22) = 255.283, *p* < .001, *η*_p_2 = 0.931, 1-*β* = 1] and the significant interaction between test condition and time [*F*(1, 22) = 4.631, *p* = .044, *η*_p_2 = 0.196, 1-*β* = 0.130]. All other factors did not lead to significant effects (see Figures 4–6). In the Go/No-Go task, response times were significantly influenced by test conditions across all analyses, with additional effects of time and test condition*time interactions in horizontal and vertical trials. No consistent main effects of group were observed. Additional analyses with multiple t-tests showed significant differences between pre-and post-test (factor time) in the VR intervention group for the sport-specific Flanker task and congruent trials [*t*(11) = −3.284, *p* < .01, *d* = 1.964, 1-*β* = 0.999] but not for the control intervention group [*t*(12) = 1.921, p = .079, *d* = 0.533, 1-*β* = 0.566]. For the incongruent trials, there are significant differences between the pre-and post-test for the VR intervention group for the sport-specific Flanker task [*t* (11) = 6.804, *p* < .01, *d* = 1.928, 1-*β* = 0.999], but again not for the control intervention group [*t*(9) = 0.684, p = .511, *d* = 0.450, 1-*β* = 0.425]. The significant interaction effect between time*group suggests a training-intervention effect on participants' performance in the corresponding tasks (see Figure 7). Follow-up t-tests revealed significant pre-post improvements in the VR intervention group for both congruent and incongruent trials of the sport-specific Flanker task. In contrast, no significant changes were found in the control group. Post-hoc power analyses confirmed very high power for the significant effects.

For the response time parameters of the Go/No go task, the results showed a significant effect of the test condition on the response times [F(1, 22) = 286.434, *p* = <.001, *η*_p_2 = 0.919, 1-β = 1]. Furthermore, results showed significant main effects also for the response time in horizontal trials by the factors time [F(1, 22) = 3.064, *p* = .003, *η*_p_2 = 0.370, 1-β = 0.299], test condition [F(1, 22) = 216.705, *p* < .001, *η*_p_2 = 0.916, 1-β = 1], the interaction test condition*group [F(1, 22) = 1.112, *p* = .050, *η*_p_2 = 0.179, 1-β = 0.121], and test condition*time [F(1, 22) = 3.624, *p* < .001, *η*_p_2 = 0.640, 1-β = 0.840] but not for time*group [F(1, 22) = 0.230, *p* = .637, *η*_p_2 = 0.208, 1-β = 0.138]. For the response times in vertical trials, the results showed significant effects for test condition [F(1, 22) = 255.283, *p* < .001, *η*_p_2 = 0.931, 1-β = 1] and the significant interaction between test condition and time [F(1, 22) = 4.631, *p* = .044, *η*_p_2 = 0.196, 1-β = 0.130]. All other factors did not lead to significant effects (see Figures 4–6). In the Go/No-Go task, response times were significantly influenced by test condition across all analyses, with additional effects of time and test condition*time interactions in horizontal and vertical trials. No consistent main effects of group were observed.

Additional analyses with multiple t-tests showed significant differences between pre-and post-test (factor time) in the VR intervention group for the sport-specific Flanker task and congruent trials [t(11) = −3.284, *p* < .01, d = 1.964, 1-β = 0.999] but not for the control intervention group [t(12) = 1.921, *p* = .079, d = 0.533, 1-β = 0.566]. For the incongruent trials, there are significant differences between the pre-and post-test for the VR intervention group for the sport-specific Flanker task [t(11) = 6.804, *p* < .01, d = 1.928, 1-β = 0.999], but again not for the control intervention group [t(9) = 0.684, *p* = .511, d = 0.450, 1-β = 0.425]. 1-β1-βThe significant interaction effect between time*group suggests a training-intervention effect on participants’ performance in the corresponding tasks (see Figure 7). Follow-up t-tests revealed significant pre–post improvements in the VR intervention group for both congruent and incongruent trials of the sport-specific Flanker task. In contrast, no significant changes were found in the control group. *post-hoc* power analyses confirmed very high power for the significant effects.

### 3.2 Accuracy

We did not find any significant effects on the accuracy parameters for the factors test condition, time, group, or any interaction (for the values, see Supplementary Table S1).

## 4 Discussion

For the current study investigated the effects of sport-specific CT with VR on the EFs, particularly inhibition, of young ice hockey players. By comparing an intervention group undergoing VR-based training with a control group receiving technical skills training, the study revealed significant improvements in inhibitory control for the VR intervention group, as measured by response times in the sport-specific Flanker task. These enhancements were observed in both congruent and incongruent conditions, demonstrating the efficacy of VR training in improving players’ ability to manage distracting stimuli and maintain focus under pressure. However, no significant changes were found in domain-general EF tasks, suggesting that the benefits of VR training may be context-dependent. These findings contribute to the understanding of how specific, immersive CT can support the development of sport-specific cognitive skills (55, 58, 59).

The results align with and extend prior research demonstrating the potential of VR-based CT. Earlier studies, such as Grosprêtre et al. (46) and Huang (45), highlighted the positive impact of VR on EFs, such as inhibition and task-switching, in general populations. However, the current study uniquely focuses on a sport-specific population, offering new insights into how VR training can replicate real-world cognitive demands. The intervention's sport-specific nature likely explains its superior efficacy compared to generic VR training approaches, supporting arguments made by Montuori et al. (54) and Scharfen & Memmert (35) about the importance of domain-specific training. The findings also resonate with the ecological validity argument emphasized by Heilmann et al. (55) and Musculus et al. (53). Unlike traditional CT methods, which often lack real-world applicability, VR allows athletes to engage in cognitively demanding tasks embedded within sport-relevant contexts. For instance, the “Find the Lane” and “Multiple-Object-Tracking” exercises in this study closely mimic in-game decision-making scenarios, facilitating the transfer of learned skills to actual performance settings. However, the absence of significant improvements in domain-general EF tasks raises questions about the generalizability of sport-specific training effects. This is consistent with the findings of Fransen (60) and Furley et al. (61), who reported limited far-transfer effects of CT. The specificity of cognitive gains highlights the need for targeted interventions tailored to the unique demands of each sport.

Accuracy results revealed no significant effects of test condition, time, group, or their interactions, indicating that the VR intervention did not influence accuracy in either sport-specific or domain-general tasks. This suggests that the benefits of VR training were primarily reflected in response speed rather than error reduction. The lack of accuracy effects aligns with previous research, suggesting that VR-based cognitive training primarily enhances processing speed rather than error reduction, likely due to ceiling effects in athletic populations.

The improvements found only in the sport-specific (ecologically valid) Flanker task but not in the domain-general EF tasks suggest that the VR intervention mainly produced near transfer effects (i.e., gains in tasks structurally similar to the trained scenarios) rather than far transfer to unrelated EF tasks. This aligns with much of the existing literature showing that far transfer is rare and harder to achieve (29, 35). The current findings have several practical implications for coaches, trainers, and sports psychologists. First, integrating sport-specific VR training into regular practice routines can provide young athletes with a competitive edge by enhancing their cognitive capabilities. This study's significant improvements in inhibitory control suggest that athletes trained in VR situations may be better equipped to make rapid decisions and adapt to complex game scenarios. Moreover, the immersive nature of VR training can increase engagement and motivation among athletes, potentially leading to better adherence to training protocols (62–64) Second, the results underscore the importance of ecological validity in cognitive assessments. Traditional EF tests, while valuable, may not fully capture the nuanced cognitive demands of specific sports. Developing and utilizing sport-specific measures ensures a more accurate evaluation of an intervention's effectiveness.

### 4.1 Limitations

While the results are promising, several limitations should be acknowledged. The small sample size (*N* = 25) limits the generalizability of the findings. Larger-scale studies are needed to confirm the efficacy of VR training across different age groups, skill levels, and sports disciplines. Moreover, the intervention duration of nine weeks may not have been sufficient to observe long-term effects or potential plateauing of cognitive improvements, especially in high-level athletes. Future research should investigate extended training periods and incorporate follow-up assessments to determine the long-term sustainability of the observed benefits. Although appropriate for the research objectives, the study's reliance on specific EF measures may not fully capture the broader range of cognitive processes influenced by VR training. Including additional metrics, such as on-ice performance indicators or neurophysiological assessments, could provide a more comprehensive understanding of the intervention's impact. Additionally, while relevant, the control group engaged in traditional technical ice hockey training, which does not allow for a direct comparison with a domain-general VR training group. Introducing a broader range of control conditions in future studies would strengthen the ability to isolate the effects of sport-specific VR training. Another potential limitation concerns the controlled lab environment in which the VR training was conducted. Although the intervention was designed to mimic game scenarios, the lack of actual on-ice conditions may limit the ecological transferability of the findings. Future research should explore ways to integrate VR training into real-world practice environments to bridge this gap. A further limitation of the present study is the absence of a standardized visual acuity test as part of the experimental protocol. Although players enrolled in the academy typically undergo regular vision screenings as part of their medical monitoring, it cannot be excluded that undetected individual differences in visual function may have influenced task performance. As the experimental tasks required acceptable visual discrimination (e.g., arrows, shapes, and hockey-specific images), reduced visual acuity could potentially confound results, particularly in relation to executive function performance. Future research should control for this factor by including a standardized visual acuity test to ensure consistent task validity.

## 5 Conclusion

This study demonstrates the effectiveness of sport-specific VR CT in enhancing inhibitory control among young ice hockey players. The significant improvements observed in ecologically valid tasks emphasize the value of tailoring cognitive interventions to the specific demands of a sport. While the findings contribute to the growing evidence supporting the use of VR for CT, they also underscore the need for further research to address the identified limitations. In conclusion, VR-based sport-specific CT represents a promising tool for athlete development, offering a unique combination of immersion, engagement, and ecological validity. By addressing the challenges of sample size, long-term effects, and real-world applicability, future studies can solidify the role of VR in optimizing athletic performance and advancing our understanding of CT in sports.

## Data Availability

The datasets presented in this study can be found in online repositories. The names of the repository/repositories and accession number(s) can be found in the article/Supplementary Material.
