# Supplementary material for: The influence of specific cognitive training in virtual reality on the inhibition of elite young ice hockey players
Source: Front Sports Act Living. 2025 Oct 30;7:1682165. doi: 10.3389/fspor.2025.1682165 (PMC12611849; doi:10.3389/fspor.2025.1682165)
Supplement: Supplementary file 1 [file Table1.docx]

Supplementary Material

# Supplementary Tables

**Table 1** Results of executive function tasks (domain-generic version)

| Flanker task | | | | | | |
| --- | --- | --- | --- | --- | --- | --- |
| Parameter | | | **Intervention** | | **Control** | |
|  |  |  | **Pre** | **Post** | **Pre** | **Post** |
| Response time [ms] | | **Incongruent** | 471.44 (114.23) | 464.65 (83.85) | 512.28 (87.41) | 482.28 (92.33) |
|  |  | **Congruent** | 446.48 (88.61) | 448.74 (80.77) | 470.63 (80.53) | 482.28 (69.99) |
|  |  | **Flanker effect** | 24.96 (41.96) | 15.91 (31.45) | 41.65 (31.95) | 39.42 (43.30) |
| Accuracy [%] | | **Incongruent** | 98.88 (3.45) | 95.49 (3.59) | 96.47 (4.28) | 94.87 (4.37) |
|  |  | **Congruent** | 98.61 (2.14) | 98.44 (2.10) | 99.04 (1.32) | 98.08 (2.38) |
| Cued Go / No go task | | | | | |  |
| Parameter | | **Intervention** | | **Control** | |  |
|  |  | **Pre** | **Post** | **Pre** | **Post** |  |
| Response time [ms] | **Mean** | 341.95 (43.37) | 338.46 (43.06) | 353.27 (64.19) | 352.75 (50.53) |  |
|  | **Vertical** | 341.81 (44.80) | 336.50 (43.45) | 351.83 (67.24) | 352.77 (52.08) |  |
|  | **Horizontal** | 342.55 (49.94) | 339.68 (48.82) | 363.28 (70.90) | 355.92 (66.81) |  |
| Accuracy [%] | **Error rate** | 2 (1.6) | 6.2 (1.3) | 2.6 (1.6) | (1.3) |  |

**Table 2** Results of executive function tasks (sport-specific version)

| Flanker task | | | | | | |
| --- | --- | --- | --- | --- | --- | --- |
| Parameter | | | **Intervention** | | **Control** | |
|  |  |  | **Pre** | **Post** | **Pre** | **Post** |
| Response time [ms] | | **Incongruent** | 752.97 (152.54) | 574.94 (110.38) | 772.50 (146.98) | 764.38 (125.82) |
|  |  | **Congruent** | 742.74 (146.35) | 561.66 (101.55) | 737.41 (153.28) | 713.38 (118.49) |
|  |  | **Flanker effect** | 10.23 (31.81) | 13.28 (22.27) | 8.13 (42.56) | 24.03 (23.43) |
| Accuracy [%] | | **Incongruent** | 97.92 (3.61) | 97.59 (3.52) | 98.33 (2.76) | 98.08 (3.51) |
|  |  | **Congruent** | 98.09 (1.80) | 96.13 (5.44) | 99.17 (1.38) | 96.96 (4.88) |
| Cued Go / No go task | | | | | |  |
| Parameter | | **Intervention** | | **Control** | |  |
|  |  | **Pre** | **Post** | **Pre** | **Post** |  |
| Response time [ms] | **Mean** | 596.91 (77.41) | 574.52 (110.23) | 659.92 (101.25) | 617.79 (84.48) |  |
|  | **Vertical** | 591.49 (80.90) | 569.25 (113.14) | 653.47 (102.70) | 613.33 (84.77) |  |
|  | **Horizontal** | 618.42 (76.58) | 594.35 (114.49) | 685.87 (108.09) | 636.73 (94.31) |  |
| Accuracy [%] | **Error rate** | 3.67 (6.37) | 1.09 (1.98) | 1.80 (1.40) | 0.92 (1.49) |  |
